# Expression of antioxidant enzymes in lesions of multiple sclerosis and its models

**DOI:** 10.1038/s41598-022-16840-w

**Published:** 2022-07-26

**Authors:** Dorsa Moezzi, Yifei Dong, Rajiv W. Jain, Brian M. Lozinski, Samira Ghorbani, Charlotte D’Mello, V. Wee Yong

**Affiliations:** Department of Clinical Neuroscience, Hotchkiss Brain Institute, University of Calgary, 3330 Hospital Drive, Calgary, AB T2N 4N1 Canada

**Keywords:** Multiple sclerosis, Diseases of the nervous system, Neuroimmunology

## Abstract

Oxidative stress promotes tissue injury in the central nervous system in neurological disorders such as multiple sclerosis (MS). To protect against this, antioxidant enzymes including superoxide dismutase-1 (SOD1), heme oxygenase-1 (HO-1), peroxiredoxin-5 (PRDX5) and glutathione peroxidase-4 (GPX4) may be upregulated. However, whether antioxidant enzyme elevation in mouse models of neurodegeneration corresponds to their expression in human diseases such as MS requires investigation. Here, we analyzed and compared the expression of SOD1, HO-1, PRDX5 and GPX4 in the murine spinal cord of three models of MS: focal lesions induced by (1) oxidized phosphatidylcholine or (2) lysophosphatidylcholine (lysolecithin), and (3) diffuse lesions of experimental autoimmune encephalomyelitis. Notably, CD68^+^ microglia/macrophages were the predominant cellular populations that expressed the highest levels of the detected antioxidant enzymes. Overall, the expression patterns of antioxidant enzymes across the models were similar. The increase of these antioxidant enzymes was corroborated in MS brain tissue using spatial RNA sequencing. Collectively, these results show that antioxidant capacity is relatively conserved between mouse models and MS lesions, and suggest a need to investigate whether the antioxidant elevation in microglia/macrophages is a protective response during oxidative injury, neurodegeneration, and MS.

## Introduction

Multiple sclerosis (MS) is a chronic inflammatory and neurodegenerative disease of the central nervous system (CNS), characterized by immune cell infiltration, demyelination and axonal injury^1^. MS leads to severe physical and neurological complications such as impaired cognition, mobility loss, pain, as well as gastrointestinal and urinary dysfunction. While the cause(s) of MS remains unclear, MS pathophysiology has been extensively studied and a key feature of MS neurodegeneration is oxidative stress^2–6^. In both relapsing and progressive MS, immune cells such as microglia and macrophages found in brain lesions are associated with markers of oxidative stress such as oxidized lipids, oxidized DNA, and malondialdehyde^7,8^. Studies in the experimental autoimmune encephalomyelitis (EAE) animal model of MS suggest that activated immune cells can promote neurodegeneration via the release of free radicals including reactive nitrogen and oxygen species^9,10^. We recently reported that oxidized phosphatidylcholines (OxPCs) found in MS brain lesions kill human neurons and oligodendrocytes in vitro and promote neurodegeneration when injected into the spinal cords of mice^7^. Together, these results suggest oxidative stress is an important mediator of neurodegeneration to overcome in MS.

In response to oxidative stress during inflammation and tissue damage, cells activate transcription factors such as nuclear factor E2–related factor 2 (Nrf2)^4^ to upregulate antioxidant proteins including superoxide dismutase (SOD), heme oxygenase (HO), peroxiredoxins (PRDX), and glutathione peroxidases (GPX). In active demyelinating MS lesions, the enhanced expression of SOD1 was detected in MHC class II positive macrophages/microglia^11^. HO-1, the inducible form of HO was found to suppress EAE progression in mice^12^. PRDX5, an enzyme with peroxynitrite reductase activity, was elevated in both the normal appearing white matter (NAWM) and lesions of MS brains compared to healthy controls^13^. Additionally, GPX4 which inhibits ferroptosis by converting lipid hydroperoxides into lipid alcohols and thereby prevents lipid peroxidation, was downregulated in the grey matter of MS patients and in spinal cord neurons during EAE^14^. This decreased level of GPX4 in the grey matter and detection of iron elevation in deep grey matter of MS lesions^15^ hint at the possibility that ferroptosis, iron-dependent cell death, may be occurring in MS lesions.

**Figure 1:**
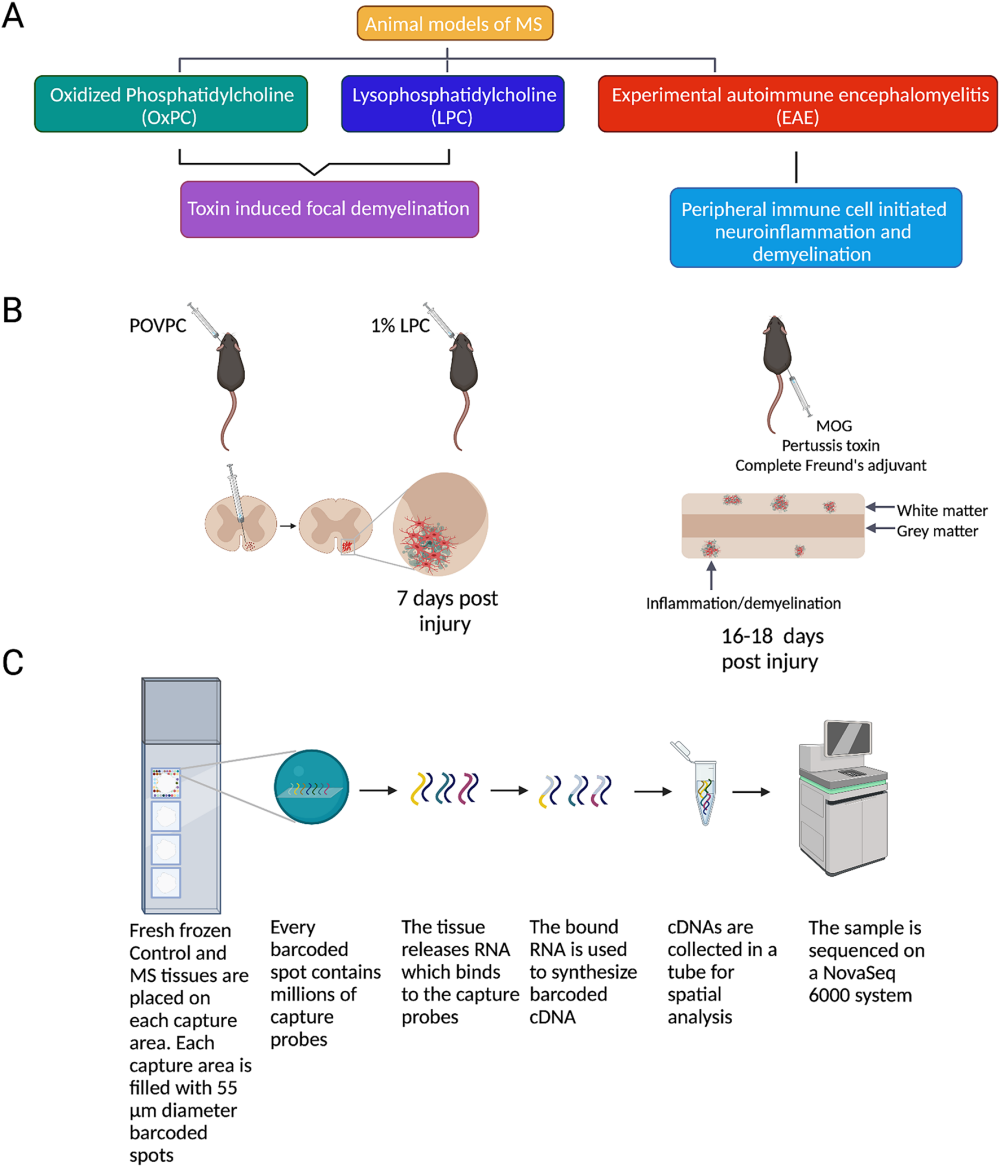
A guide to tissue collection and analysis. (**A**–**B**) Schematic diagram of three animal models of MS: Experimental autoimmune encephalomyelitis (EAE), Lysophosphatidylcholine (LPC, lysolecithin), and Oxidized phosphatidylcholine (OxPC). (**C**) Schematic diagram of human spatial RNA sequencing experiment.

The above studies suggest the importance of antioxidant enzymes in MS. However, it is unclear which cells in the CNS express these enzymes and whether their expression in various MS models is representative of human MS lesions. To address these questions, we aimed to compare the tissue and cellular expression of SOD1, HO-1, GPX4, and PRDX5 between MS samples and multiple mouse models of MS. The latter included focal spinal cord lesions induced by oxidized phosphatidylcholines (OxPCs)^7^ and lysophosphatidylcholine (LPC, also referred to as lysolecithin)^16^, as well as EAE (Fig. 1A–B).

## Results

### Antioxidant enzymes accumulate in OxPC injected lesions

**Figure 2:**
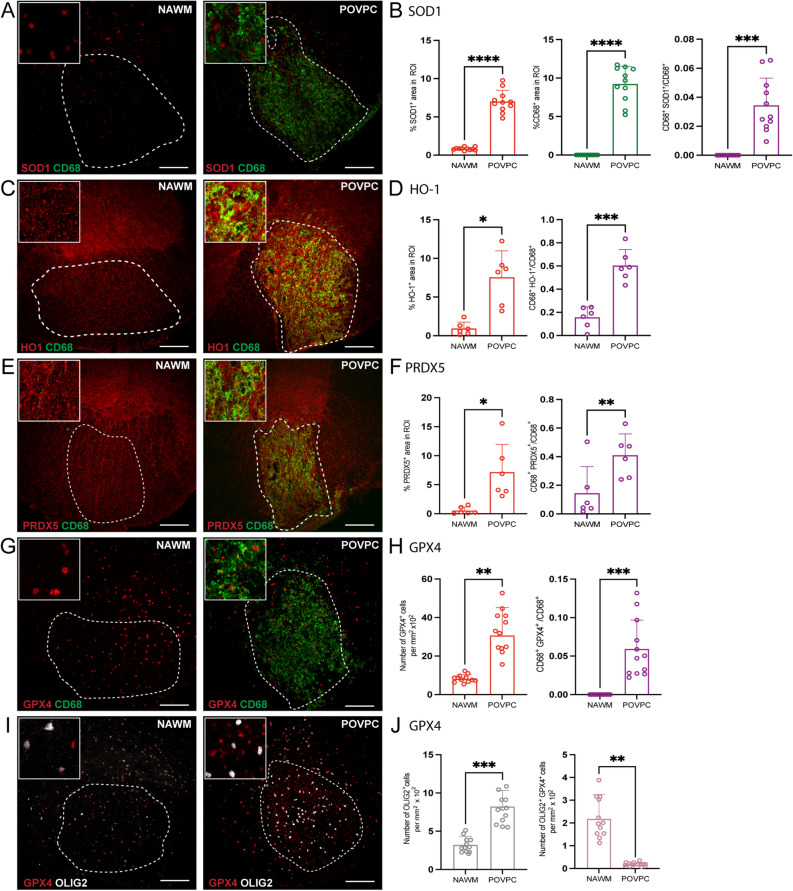
Expression of antioxidant enzymes is upregulated in POVPC-induced spinal cord lesions. (**A**, **C**, **E**, **G**, **I**) Representative confocal images of NAWM or POVPC injected spinal cord labeled with CD68 for microglia/macrophage (green), antioxidant enzyme of interest (red), and OLIG2 for oligodendrocyte lineage cells (white). Dotted line indicates the NAWM or lesion region of interest (ROI) selected for image analysis. (**B**, **D**, **F**) Bar graphs comparing the percent of ROI that is CD68^+^, antioxidant enzyme of interest, and CD68^+^ antioxidant enzyme^+^. (**H**) Bar graph comparing the number of GPX4^+^ cells per mm^2^ × 10^2^ of ROI and percent of ROI that is CD68^+^ GPX4^+^ (**J**) Bar graph representing number of OLIG2 + cells per mm^2^ × 10^2^ of ROI and percent of ROI that is OLIG2^+^ GPX4^+^. Scale bar = 100 µm. Data are shown as mean ± S.D, n = 6–12 mice. Significance indicated as *p < 0.05, **p < 0.01, ***p < 0.001, ****p < 0.0001, two-tailed, paired student’s t-test.

Spinal cords from mice injected with OxPC were isolated after 7 days and the expression of antioxidant enzymes SOD1, HO-1, PRDX5 and GPX4 was detected and quantified by confocal immunofluorescence microscopy analysis. The contralateral normal appearing white matter (NAWM) was not inflamed as defined by the lack of CD68^+^ cells accumulation (Fig. 2A, C, E, G). In contrast, the white matter containing OxPC lesion had substantial cellular changes (Fig. 2B, D, F, H) indicative of injury that we previously described^7^. The OxPC lesion had increased SOD1, HO-1, PRDX5 and GPX4 immunoreactivity (Fig. 2A–H) and accumulation of CD68^+^ microglia/macrophages relative to NAWM. There was also a significant increase in the proportion of CD68^+^ microglia/macrophages that was associated with these antioxidant enzymes (Fig. 2A–H).

For GFAP^+^ astrocytes, there was a loss of cell number within the OxPC lesion compared to NAWM, as previously noted^7^. However, the proportion of GFAP^+^ cells expressing SOD1^+^, HO-1^+^ or PRDX5^+^ did not change (Supplementary Fig. 1B, D and F).

In the NAWM, GPX4 was mostly associated with OLIG2^+^ cells (Fig. 2I–J). However, while the density of OLIG2^+^ cells increased in OxPC lesions compared to the NAWM, the amount of GPX4 immunoreactivity that was associated with OLIG2^+^ cells decreased (Fig. 2I–J), indicating that this increase of GPX4 was not from infiltrating OLIG2^+^ cells but was from CD68^+^ microglia/macrophages.

### Antioxidant enzyme levels in lysophosphatidylcholine (LPC) model of demyelination and neurodegeneration

**Figure 3:**
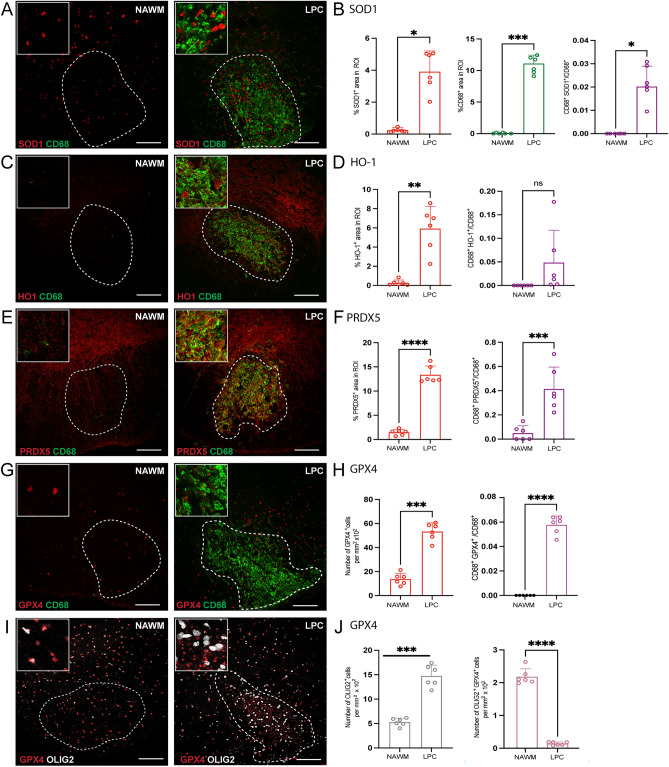
Expression of antioxidant enzymes is elevated in LPC-induced lesions. (**A**, **C**, **E**, **G**, **I**) Representative confocal images of NAWM or LPC injected mice labeled with CD68 for microglia/macrophage (green), antioxidant enzyme of interest (red), OLIG2 for oligodendrocyte lineage cells (white). Dotted line indicates the lesion ROI selected for image analysis. (**B**, **D**, **F**) Bar graphs comparing the percent of ROI that is CD68^+^, antioxidant enzyme of interest, and CD68^+^ antioxidant enzyme^+^. (**H**) Bar graph comparing the number of GPX4^+^ cells per mm^2^ × 10^2^ of ROI and percent of ROI that is CD68^+^ GPX4^+^ (**J**) Bar graph representing number of OLIG2 + cells per mm^2^ × 10^2^ of ROI and percent of ROI that is OLIG2^+^ GPX4^+^. Scale bar = 100 µm. Data are shown as mean ± S.D, n = 6 mice. Significance indicated as *p < 0.05, **p < 0.01, ***p < 0.001, ****p < 0.0001, two-tailed, paired student’s t-test.

LPC is a detergent which causes demyelination and neurodegeneration when injected into the CNS white matter^17,18^. The expression of GPX4, PRDX5 and HO-1 was determined from spinals cords 7 days after LPC injection. Similar to OxPC lesions, LPC injected spinal cord white matter had prominent accumulation of CD68^+^ microglia/macrophage, and elevated SOD1, HO-1, PRDX5 and GPX4 (Fig. 3A–H) in the lesion compared to the NAWM control. As well, there was a significant increase in the proportion of CD68^+^ cells positive for SOD1^+^, PRDX5^+^, and GPX4^+^ in the LPC lesions (Fig. 3B, F, and H). While the proportion of CD68^+^ HO-1^+^ cells was not significantly different from NAWM, there was a trend of increase in the lesion (Fig. 3D).

While GFAP^+^ cells were decreased within the LPC lesion as a result of cell death^17^, the proportions of GFAP^+^ cells expressing SOD1^+^ or PRDX5^+^ increased in LPC induced lesions compared to control (Supplementary Fig. 2A–B, E–F). In contrast, there was no difference in proportion of GFAP^+^ HO-1^+^ cells (Supplementary Fig. 2C–D).

There was an elevation of OLIG2^+^ cells in the day 7 LPC lesions compared to NAWM; this is consistent with repopulation of oligodendrocyte lineage cells after demyelination that we have previously described^19^. Similar to OxPC lesions, many of the OLIG2^+^ cells did not express detectable GPX4^+^ within the LPC lesion (Fig. 3I–J).

### Antioxidant enzyme levels in EAE

**Figure 4:**
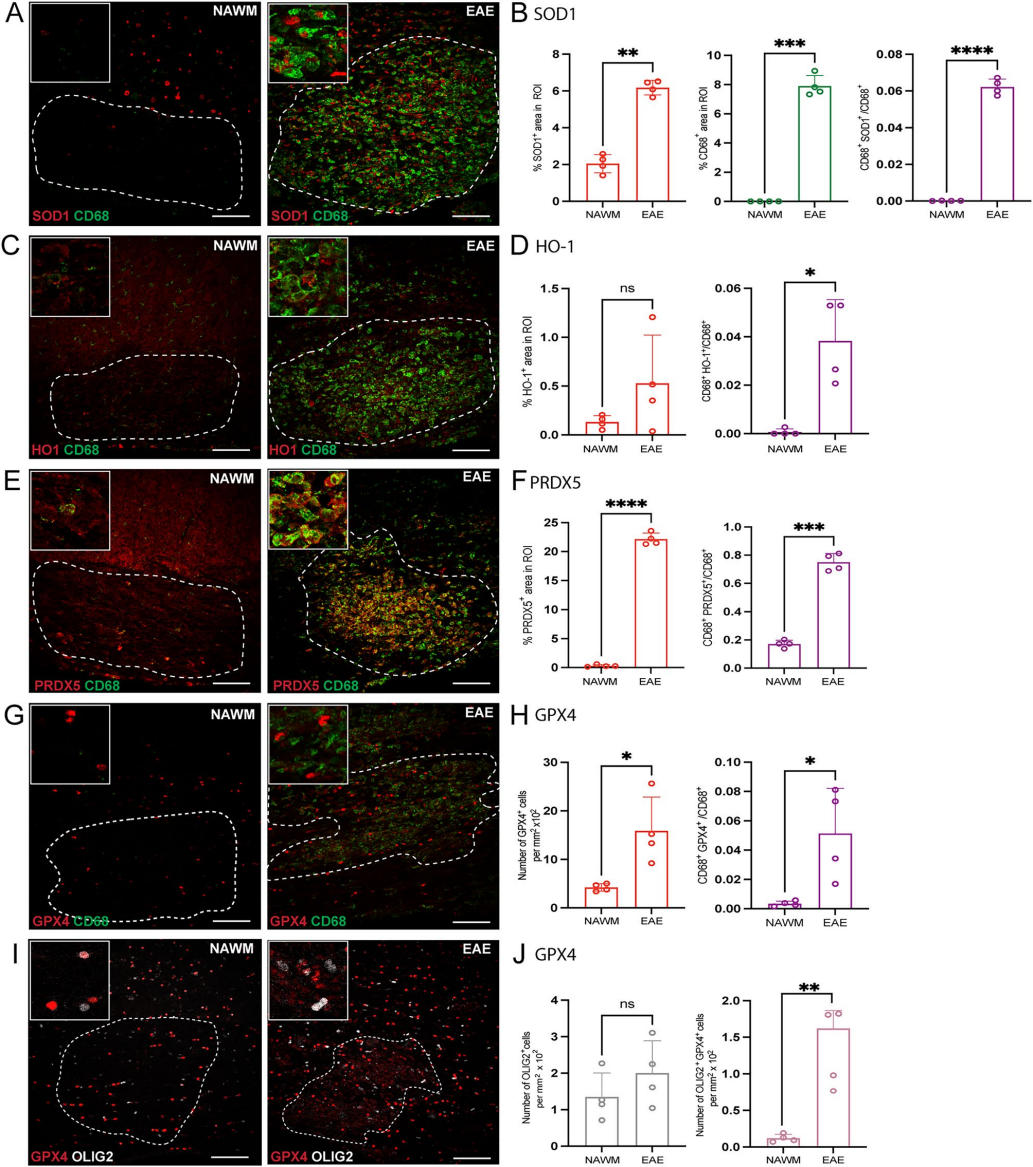
Detection of antioxidant enzymes is increased in EAE-induced lesions. (**A**, **C**, **E**, **G**, **I**) Representative confocal images of NAWM or EAE-induced mice labeled with CD68 for microglia/macrophage (green), antioxidant enzyme of interest (red), OLIG2 for oligodendrocyte lineage cells (white). Dotted line indicates the lesion ROI selected for image analysis. (**B**, **D**, **F**) Bar graphs comparing the percent of ROI that is CD68^+^, antioxidant enzyme of interest, and CD68^+^ antioxidant enzyme^+^. (**H**) Bar graph comparing the number of GPX4^+^ cells per mm^2^ × 10^2^ of ROI and percent of ROI that is CD68^+^ GPX4^+^ (**J**) Bar graph representing number of OLIG2 + cells per mm^2^ × 10^2^ of ROI and percent of ROI that is OLIG2^+^ GPX4^+^. Scale bar = 100 µm. Data are shown as mean ± S.D, n = 4 mice. Significance indicated as *p < 0.05, **p < 0.01, ***p < 0.001, ****p < 0.0001, two-tailed, paired student’s t-test.

Mice immunized with myelin peptides such as myelin oligodendrocyte glycoprotein (MOG) are used to model widespread neuroinflammation in MS^20^. To determine if antioxidant enzymes were also upregulated in EAE, the expression of SOD1, HO-1, PRDX5 and GPX4 was analyzed in spinal cords from mice with peak EAE disability score. Confocal immunofluorescence microscopy analysis was used to compare NAWM with inflamed EAE lesions which had increase of CD68^+^ cells (Fig. 4A–B). The expression of SOD1, PRDX5, and GPX4 was significantly increased in the lesion; while the level of HO-1 had an increasing trend, it was not significant (Fig. 4A–J). Additionally, the proportion of CD68^+^ SOD1^+^, CD68^+^ HO-1^+^, CD68^+^ PRDX5^+^, and CD68^+^ GPX4^+^ cells was significantly elevated in EAE lesions (Fig. 4B, D, F, H).

Unlike OxPC and LPC lesions, there was no apparent loss of GFAP^+^ astrocytes in the EAE lesions compared to NAWM (Supplementary Fig. 3A–B). In addition, the percent GFAP^+^ SOD1^+^ cells decreased, the percent GFAP^+^ HO-1^+^ cells remained unchanged, and the percent GFAP^+^ PRDX5^+^ significantly increased in lesions (Supplementary Fig. 3B, D, and F). Moreover, unlike OxPC and LPC focal lesions, the number of OLIG2^+^ cells did not change in EAE lesions while the proportion of OLIG2^+^ GPX4^+^ cells was elevated (Fig. 4I–J).

### Spatial RNA sequencing reveals upregulation of Nrf2 and antioxidant transcripts in MS brain lesions

**Figure 5:**
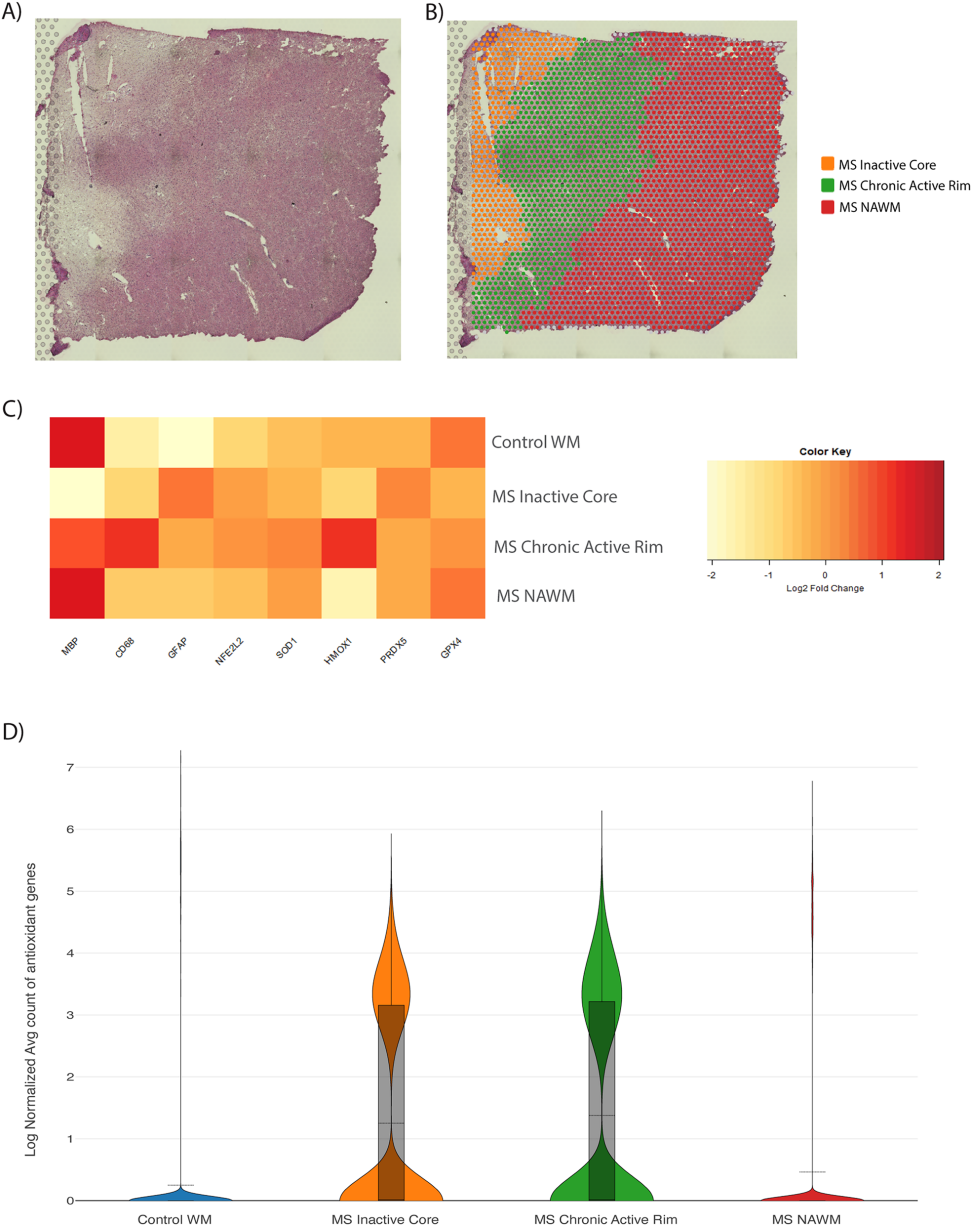
Spatial transcriptomic analysis of antioxidant protein expression in chronic active MS brain lesions. (**A**) Representative microscopy image of post-mortem MS brain tissue labeled with hematoxylin and eosin. This is a white matter region of the frontal lobe. (**B**) Spatial demarcation of the MS tissue into anatomical regions (NAWM, inactive core, and active edge) based on their expression of *MBP* and cellularity. (**C**) Heatmap showing the expression of genes across control WM and the segmented regions of MS tissue. (**D**) Violin plot showing the combined sum of expression of *SOD1*, *HMOX-1*, *PRDX5*, and *GPX4* in the different anatomical regions of the MS tissue. Data representative from 2 healthy control and 2 MS samples.

To assess if SOD1, HO-1, PRDX5, and GPX4 was also associated with and elevated in MS lesions, we performed spatial RNA sequencing analysis, as shown in Fig. 1C, on post-mortem non-neurological disease controls and MS brain tissue sections containing NAWM, an inactive core of a lesion, and its chronic active rim (Fig. 5A). In comparison with non-MS control WM, tissues from MS brains contain demyelinated white matter lesions as indicated by the absence (for inactive core) or reduction (for chronic active rim) of myelin as shown by hematoxylin and eosin histological stains (Fig. 5A). The inactive core, chronic active rim and NAWM associated with MS lesions were also spatially separated based on their expression of myelin basic protein (MBP) transcripts (Fig. 5B), cellularity (not shown) and MBP/CD45 immunofluorescence (Supplementary Fig. 4). By normalized relative expression for spatial RNA transcripts, we found an increase in expression of CD68 at the active edge and increased GFAP in the inactive core of lesion consistent with the localization of microglia/macrophage in chronic active rims validating our segmentation (Fig. 5C)^21,22^. The low and intermediate levels of MBP transcripts in the inactive core and chronic active rim, respectively, compared to NAWM or control WM further corroborated our segmentation (Fig. 5C). Expression of Nrf2, a transcription factor that induces the transcription of antioxidant enzymes, trended towards an increase in the chronic active rim and inactive core relative to NAWM and control WM (Fig. 5C) suggesting a response against oxidative stress across MS lesions. Consistent with this, there was a trend towards elevated *SOD1* and *HO-1* transcripts in the chronic active rim region compared to the control WM and NAWM, and *PRDX5* trended towards upregulation in the inactive core region. Similar to the loss of GPX4 by OLIG2^+^ cells in the OxPC and LPC lesions, there was a trend towards *GPX4* reduction in the inactive core and chronic active rim of the MS lesions compared to the control WM (Fig. 5C). However, transcriptional activity in the inactive core and chronic active rim was higher than in NAWM and control WM resulting in an overall increase in the number of antioxidant transcripts in MS lesions (Fig. 5D). Collectively, these results show an increased expression of antioxidant enzyme mRNA across the inactive core and chronic active rim of MS lesions compared to NAWM.

**Figure 6:**
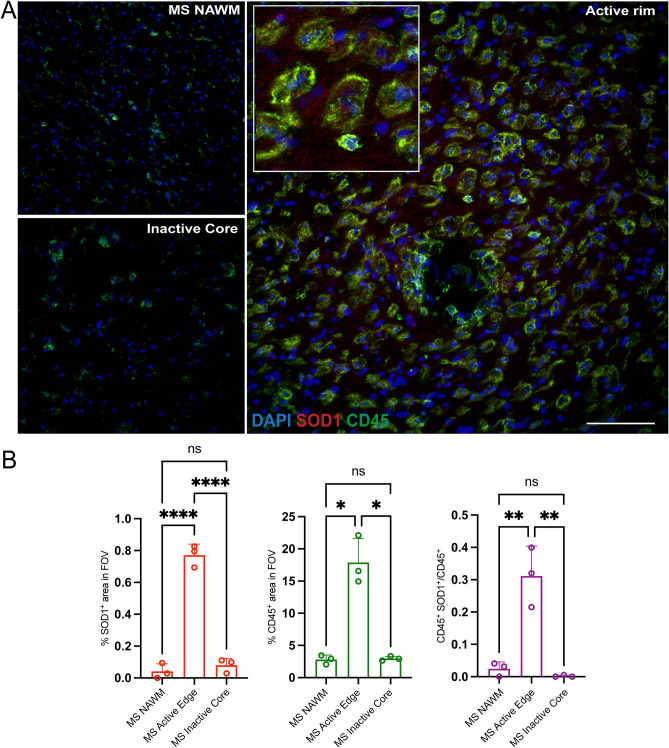
Expression of SOD1 antioxidant enzyme is elevated in the chronic active rim of MS lesions. (**A**) Representative confocal images of NAWM, inactive Core, and active rim of an MS lesion labeled with CD45 for immune cells (green), SOD1 (red) and DAPI (blue). (**B**) Bar graphs comparing the percent of field of view (FOV) that is CD45^+^, SOD1^+^, and CD45^+^SOD1^+^. Scale bar = 100 µm. Data are shown as mean ± S.D, n = 3 MS patients. Significance indicated as *p < 0.05, **p < 0.01, ***p < 0.001, ****p < 0.0001, One-way ANOVA with Tukey's post-hoc test.

To further investigate the antioxidant response in MS, the protein expression of antioxidant enzymes and CD45 in lesions of three MS cases were quantified by confocal immunofluorescence microscopy analysis. Consistent with spRNAseq analysis, the detection level of CD45^+^ cells was highest in the chronic active rim compared to the inactive core and NAWM (Fig. 6A–B). While HO-1, PRDX5 and GPX4 stainings had high background and were difficult to evaluate, SOD1 was detected. The chronic active rim had increased SOD1 immunoreactivity relative to the inactive core and NAWM (Fig. 6B). Additionally, the proportion of CD45^+^ SOD1^+^ cells increased in the chronic active rim while there was no difference detected in inactive core and NAWM (Fig. 6B).

## Discussion

The contribution of oxidative stress in producing lesions in MS has been well reviewed. Van Horssen et al.^11^ first reported that markers of oxidative injury were elevated in active demyelinating lesions while Nikic et al.^9^ found that reactive oxygen and nitrogen species produced by microglia/macrophages resulted in focal axonal degeneration. Fischer et al.^23^ reported that markers of oxidative stress were correlated with oligodendrocyte and neuronal injury, including damaged axons and dendrites. A comparison of microarray data of brain specimens concluded that oxidative stress was more pronounced in progressive stages of MS when compared to lesions of other chronic inflammatory diseases (tuberculous meningitis) or Alzheimer’s disease^23^. Thus, understanding endogenous CNS antioxidant sources may lead to new approaches to reduce oxidative stress in MS.

Here, we analyzed the expression of the antioxidant enzymes SOD1, HO-1, PRDX5, and GPX4 in MS brain lesions and mouse models of MS as well as evaluated their cellular sources in mouse spinal cord lesions. While all four antioxidant enzymes were increased in the OxPC and LPC spinal cord white matter lesions, only SOD1, PRDX5, and GPX4 were significantly elevated in the EAE spinal cord lesions (Table 1). CD68^+^ microglia/macrophages in OxPC and EAE lesions prominently upregulated all four enzymes. In contrast, HO-1 in CD68^+^ microglia/macrophages was not significantly altered in LPC lesions. Conversely, GFAP^+^ astrocytes appeared not to be involved in the production of the tested antioxidant enzymes in OxPC lesions and only elevated SOD1 and PRDX5 in LPC and EAE lesions, but not HO-1. Previously, HO-1 has been described to be mainly expressed by astrocytes in active MS lesions^11^. However, after LPC and POVPC injection, astrocytes are depleted from the center of the lesion due to the nature of the injury^7,17^, but the remaining astrocytes on the border of the lesions show a trend of increase of HO-1 after quantification. Collectively, we demonstrate the expression of antioxidant enzymes in OxPC, LPC, or EAE lesions in microglia/macrophages in particular.

**Table 1:** Summary of changes in the 3 animal models of MS.

	POVPC	LPC	EAE
CD68	Upregulated	Upregulated	Upregulated
OLIG2	Upregulated	Upregulated	No change
GFAP	Downregulated	Downregulated	No change
SOD1	Upregulated	Upregulated	Upregulated
CD68^+^ SOD1^+^	Upregulated	Upregulated	Upregulated
GFAP^+^ SOD1^+^	No change	Upregulated	Downregulated
HO-1	Upregulated	Upregulated	No change
CD68^+^ HO-1^+^	Upregulated	No change	Upregulated
GFAP^+^ HO-1^+^	No change	No change	No change
PRDX5	Upregulated	Upregulated	Upregulated
CD68^+^ PRDX5^+^	Upregulated	Upregulated	Upregulated
GFAP^+^ PRDX5^+^	No change	Upregulated	Upregulated
GPX4	Upregulated	Upregulated	Upregulated
CD68^+^ GPX4^+^	Upregulated	Upregulated	Upregulated
OLIG2^+^ GPX4^+^	Downregulated	Downregulated	Upregulated

This table lists the changes in the level of CD68, OLIG2, and GFAP positive cells, and their expression of particular antioxidant enzymes in the three animal models of MS.

Our results are consistent with available reports in the literature. MS lesions have increased PRDX5 expression compared to control and NAWM^13^. SOD1 and HO-1 are elevated in MS lesions that are actively demyelinating, presumably as an adaptive response to the oxidative stress being encountered. Similar to our results, the mRNA of all three GPX4 mRNA isoforms were reduced in chronic MS lesions^14^. The expression of GPX4 protein levels in MS remains unclear.

We noted that GPX4 was upregulated in mouse spinal cord lesions and localized to DAPI^+^ nuclei, suggesting only the nuclear isoform was detected. Interestingly, the morphology of GPX4 within the lesions was different than in NAWM. DAPI^+^ GPX4^+^ particles in NAWM were large spheres with high immunofluorescence and evenly distributed in the tissue. Conversely, GPX4 particles in spinal cord lesions appear as smaller profiles with lower immunofluorescence, suggesting that GPX4 in the lesion may be associated with other cellular sources. The morphological and lower immunoreactivity of GPX4 is consistent with previous results that show GPX4 downregulation in MS lesion^14^. However, GPX4 exists in nuclear, cytoplasmic, and mitochondrial isoforms. Here we detect the nuclear isoform but not the mitochondrial or cytoplasmic forms. How GPX4 expression changes in response to neuroinflammation and neurodegeneration require additional investigation. Nevertheless, if GPX4 is lowered in MS lesions, and given GPX4 has important roles in preventing cell death from oxidative stress and ferroptosis^24^, therapeutic GPX4 elevation may be neuroprotective.

There are limitations in the present study. There are different stages of an MS lesion (active, chronic active, inactive) and each may express a unique antioxidant profile. In our animal models, we analyzed the peak of inflammation following OxPC, LPC, or EAE challenge in the spinal cord. Thus, future studies should consider different times of disease evolution such as early inflammation or later remyelination phases of LPC; this will contribute a better understanding on the roles of antioxidant enzymes. In addition, other antioxidants such as SOD3 or GPX7^25^ may be increased during neuroinflammation and neurodegeneration and were not addressed in our study.

Our results demonstrate localization of antioxidant enzymes within microglia and macrophages, and to a lesser extent in GFAP^+^ and OLIG2^+^ cells. This validates that microglia and macrophages are equipped to protect the CNS against oxidative damage. Although it has been reported in numerous studies that the persistent activation of microglia in MS is likely to be detrimental^9,26^, here we show the potential beneficial capacity of microglia and macrophages to neutralize the oxidative stress existent in the lesion through the increase of antioxidant enzymes.

In summary, we present evidence that several antioxidant enzymes are upregulated in different models of MS and highlight their predominant expression in microglia/macrophages during oxidative injury. The expression of these antioxidant enzymes in MS tissue are mostly elevated as well, at least at the transcript level. Understanding the antioxidant capacity of different CNS cells allows us to explain their protective role against oxidative stress and can help in the development of therapeutic strategies against neurodegeneration and MS progression.

## Methods

### Mice

Animal experiments were conducted with ethics approval from the University of Calgary Animal Care Committee. All manipulations were in accordance with regulations of the Canadian Council of Animal Care and the ARRIVE guidelines (https://arriveguidelines.org). For all the experiments, female C57Bl/6 mice from Charles River were used at the age of 6–12 weeks.

### MS specimens

The UK Multiple Sclerosis Tissue Bank at Imperial College, London (www.ukmstissuebank.imperial.ac.uk) kindly provided post-mortem frozen brain tissue from healthy control individuals and patients with progressive MS. MS brain tissues for spRNAseq was kindly provided by Dr. Alex Prat, University of Montreal, Canada. The use of the MS specimens is approved by The Conjoint Health Research Ethics Board at the University of Calgary. For lesion identification, the MS tissues from spRNAseq were characterized through LFB and H&E staining. Additionally, lesional staging was determined using MBP and CD45 staining.

### Post-mortem MS specimens for spRNAseq

The Visium Spatial Gene Expression platform (10X Genomics) was used to perform SpRNAseq. To determine optimal tissue permeabilization time, a Visium spatial tissue optimization kit was used. This demonstrated an optimum permeabilization time of 18 min. For the Visium spatial gene expression assay, 10 µm tissue sections from two frozen non-neurological disease control tissue (CO36 and CO54) and two MS tissue from secondary progressive MS patients (MS AB172, MS AB200) were placed on capture areas on Visium slides. Images of H&E stained sections were captured using a EVOS FL Auto Imaging System (ThermoFisher) with a 10× objective. Gene expression libraries were prepared as per the user guide.

### SpRNAseq analysis

The number of reads obtained from the 4 libraries ranged from 1.2–1.4 × 106. From the control tissue, an average of 45,000–78,000 mean reads/spot (1773–3071 tissue spots) and ~ 373 median genes/spot were recovered. For the MS tissue, an average of 37,000–39,000 mean reads/spot (3200–3500 tissue spots) and 200–304 median genes/spot were recovered.The Space Ranger software v.1.2 STAR using v.2.5.1 for genome alignment was utilized to process the base call (BCL) files and histology images against the GRCh38 human reference dataset. Following this, the generated count files for each library were then aggregated with normalization set to ‘Mapped’. Then, the Loupe Browser was used to visualize the aggregated cloupe file. To visualize the spatial gene expression data from human MS tissue, the Loupe Browser 5.01 (10X Genomics) was used. For healthy control tissues, H&E stains in combination with detected MBP and PLP1 transcripts were used to define areas of white matter and exclude grey matter. For MS samples, H&E in combination with detected MBP and PLP1 transcripts were used to define the areas of the inactive core and active edge; this was corroborated with CD45/MBP immunofluorescence. The density of nuclei from H&E and CD68 transcripts, and CD45/MBP immunofluorescence, were used to define the borders of the active edge and NAWM. Individual spots on the borders of distinct regions were distributed to anatomical regions based on transcriptional similarity and on their co-localization to distinct transcriptional clusters identified on a UMAP of the inactive core, active edge, and NAWM of the MS lesions. Expression of differentially expressed genes between the Control WM, MS NAWM, chronic active rim and inactive core were completed using Loupe browser. Heatmaps were generated from log(two) fold change values from Loupe browser and plotted in R using the heatmap.2 function. Violin plots were generated in Loupe browser using the normalized sum of expression of HMOX1, SOD1, GPX4, and PRDX5.

### Spinal cord surgery

The procedure for OxPC and LPC spinal cord injection was adapted from LPC demyelination experiments as described previously ^7,27^. First, to anesthetize mice, 100 mg/kg of ketamine and 10 mg/kg of xylazine were injected intraperitoneally. Buprenorphine (0.05 mg/kg) was also injected subcutaneously immediately prior to surgery and 12 to 16 h post-surgery as an analgesic. Ophthalmic gel was applied to both eyes to keep them moist throughout the surgery and recovery period. Once the mouse was fully anesthetized, the back (from ears to mid back) was shaved and disinfected with 70% ethanol and iodine solution. A scalpel was used to vertically incise the midline of the dorsal skin. Two fine point forceps were used to expose and separate the adipose and the muscle on the vertebral column. A retractor was then used to expose the protuberance of T2 thoracic vertebra, which was used as an anatomical landmark to identify the intervertebral space between T3 and T4. Once the space was identified, spring scissors were used to carefully remove the tissue and muscle between T3 and T4 without damaging the blood vessels around the target area. Before injecting the needle into the space, the meninges between T3 and T4 was removed using a 30-gauge needle. To induce the OxPC injury in the OxPC model, 0.5 µl of purified 1-O-palmitoyl-2-O-(5-oxovaleroyl)-sn-glycero-3-phosphocholine (POVPC Avanti Polar Lipids, 870606P) resuspended in PBS at 10 mg/ml was injected at a rate of 0.25 µl/min over 2 min using a 10 µl Hamilton syringe attached for a 34-gauge needle inserted 1.3 mm into the spinal cord. Focal LPC demyelination was similarly induced but with 5 µl of 1% LPC (Sigma L1381). To prevent fluid back flow after the injection, the needle was left in place for 2 min. Finally, the adipose tissue and the skin were sutured, and more ophthalmic gel was applied to both eyes to keep them moist during the recovery stage.

### Spinal cord tissue isolation

POVPC and LPC injected animals were euthanized with ketamine and xylazine overdose 7 days post-surgery. Using scissors, an incision was made into the right atrium to allow the blood outflow. Then, 12 ml of PBS followed by 12 ml of 4% paraformaldehyde in PBS was slowly injected into the left ventricle via 25-gauge needle. The lower cervical regions to the end of thoracic regions of the spinal cord was dissected from the back of the mouse and incubated in 4% paraformaldehyde in PBS at 4 °C for fixation for 24 h. The following day, the cords were transferred to tubes containing 30% sucrose solution at 4 °C for at least 72 h. Finally, the spinal cords were frozen in FSC 22 Frozen Section Media (Leica). Using a cryostat (ThermoFisher Scientific), spinal cord tissue was cut coronally into 20 µm sections, collected on to Superfrost Plus microscope slides (VWR) and stored at − 20 °C prior to immunostaining and analysis.

### EAE and EAE tissue isolation

Female C57BL/6 mice (8–10 weeks old) from Jackson Laboratories were immunized subcutaneously with MOG 35–55 peptide (50 μg/100 μl, Protein and Nucleic acid facility, Stanford University School of Medicine, Stanford, CA) emulsified in complete Freund’s adjuvant (CFA) (Thermo Fisher Scientific) containing 10 mg/ml of heat inactivated *Mycobacterium tuberculosis* H37RA (Sigma-Aldrich). 50 μl emulsion was injected at one site into each hind flank. On the day of immunization and 48 h later, animals received intraperitoneal injections of 300 ng of pertussis toxin (List Biological Laboratories). Clinical signs and physical disability of the EAE mice were evaluated daily using a 0-to-15-point scoring scale previously described^28^. To assess acute lesions, spinal cord tissues were dissected from EAE mice at the peak of clinical severity (Day 16–18 post-immunization). Following the euthanization of mice with intraperitoneal injection of ketamine (100 mg/kg) and xylazine (10 mg/kg), PBS-perfusion was performed through the left ventricle of the heart. A small incision was made at the right atrium for blood outflow and the left ventricle was carefully punctured with a 25-gauge needle to slowly inject 12 ml of PBS. Then, the spinal cords were processed as stated in spinal cord tissue isolation section. Frozen cords were cut into 20 µm longitudinal sections by a cryostat (ThermoFisher Scientific) collected on to Superfrost Plus microscope slides (VWR) and stored at − 20 °C prior to immunostaining.

### Antibodies

Primary antibodies used for immunofluorescence staining are as follows: rat anti-mouse CD68 (1:1000 of 0.5 mg/ml stcok, Biolegend, 137,002), rabbit polyclonal anti-superoxide dismutase 1 (1:200 of 0.47 mg/ml, Thermofisher, PA5-27240), rabbit monoclonal recombinant anti-heme oxygenase 1 (1:200 of 0.05 mg/ml, Abcam, ab68477), rabbit polyclonal peroxiredoxin-5 (1:200 of 0.5 mg/ml, Thermofisher, PA5-98085), rabbit recombinant monoclonal glutathione peroxidase-4 antibody (1:200 of 0.5 mg/ml, Abcam, ab125066), chicken anti-mouse GFAP IgY antibody (1:1000 of 0.5 mg/ml, Biolegend, 829401), goat anti-mouse OLIG2 (1:200 of 0.2 mg/ml, R&D systems, AF2418), and rat anti-human CD45 (1:200 of 1 mg/ml, Thermofisher, MA5-17687). All the secondary antibodies that were used were from Jackson ImmunoResearch and are as follows: Alexa Fluor 488 donkey anti-mouse IgG, Alexa Fluor 488 donkey anti-rat IgG, Alexa Fluor 488 donkey anti-goat IgG, Alexa Flour 647 donkey anti-rabbit IgG, and Cyanine Cy3 donkey anti-chicken IgY.

### Confocal immunofluorescence microscopy

Frozen slides for OxPC and LPC tissues were warmed to room temperature (RT) for 30 min and rehydrated with PBS for 10 min. For permeabilization, slides were covered with 0.2% Triton X-100 in PBS for 10 min. Slides were then blocked with the mixture of horse blocking buffer (PBS, 10% horse serum, 1% BSA, 0.1% Triton X-100, 0.1% cold fish stain gelation, and 0.05% Tween-20) for 1 h at RT. Sections were then incubated with primary antibodies resuspended in an antibody dilution buffer (PBS, 1% BSA, 0.1% cold fish stain gelation, 0.1% Triton X-100) overnight at 4 °C. The next morning, slides were washed with PBS containing 0.05% Tween-20 three times, 5 min each. Once the excess fluid was removed from the slides, they were then covered with species-specific secondary antibodies and 1 µg/ml of DAPI resuspended in the antibody dilution buffer for 1 h at RT. After another three 5-min washes with PBS containing 0.05% Tween-20, slides were covered by fluoromount-G medium (SouthenBiotech) and kept in a dark area to avoid light exposure. For EAE tissues, slides were warmed to RT for 30 min, fixed with 4% paraformaldehyde for 15 min, and then washed once with PBS. All remaining steps were the same as above. All the immunofluorescence stains were accompanied by a slide stained with only secondary antibodies and DAPI to confirm the specificity of secondary antibodies that were used. Fluorescent preparations were imaged at RT using a Leica TCS SP8 laser confocal microscope, using 25 × 0.5 NA water objective for all the samples. During imaging, to excite the fluorophores from antibodies bound to samples, the 405, 488, 552, and 640 nm lasers were utilized. The settings were set up to use two low dark current Hamamatsu PMT detectors and two high sensitivity hybrid detectors on the SP8. Images of samples were acquired in z-stack, 1 airy unit pinhole, 0.75 × zoom, and 0.57 µm per optical section and 2048 × 2048 pixels xy resolution. Consistent laser, gain, and offset settings to maximize contrast and minimize saturation were adjusted equally for all samples within each set of experiments. For mice with spinal cord injections, one lesion epicenter was acquired per biological replicate for analysis. For mice with EAE, three to four representative lesion areas were acquired and averaged per each biological replicate for analysis. Leica Application Suite X was used for image acquisition and ImageJ was used for image threshold and particle analysis.

### Confocal image analysis

For all the acquired images, the maximum intensity projections were created, the channels were separated, and their TIF files were saved. A region of interest (ROI) was drawn around a lesion based on CD68 labelling. A ROI equivalent to the size of lesion was also drawn in NAWM region a few sections away from the start of the lesion and the area outside the ROI was cleared. Images were then converted from 8-bit to RGB, and the color threshold was used to determine the positive signal. The chosen color threshold value was checked with both the secondary control and control images to eliminate the non-specific background. The threshold, size and circularity of the particles remained consistent throughout the imaging for each experimental set. To determine CD68^+^ antioxidant enzyme^+^ the threshold for CD68^+^ cells was determined, and the quantification results were recorded. Then, the threshold was overlaid with the antioxidant channel, the outside was cleared, and the threshold setting was applied for that channel. The quantifications were recorded and utilized for further analysis using GraphPad Prism8 software. One representative image for each data set was selected where the maximum intensity projection of each channel in a z-stack was displayed in pseudo color in ImageJ. Only the brightness and contrast settings for each channel were adjusted for better presentation. The scale bar for main and supplementary figures = 100 µm.

### Statistics

Analysis and Graphs were generated using GraphPad Prism 9 (LaJolla, CA). Data shown are the individual data points where each point on a bar graph represents a biological replicate and mean ± standard deviation. To analyze statistically significant differences between the two groups (NAWM vs lesion), two-tailed, paired t-test was used. To analyze statistically significant differences between the three groups (NAWM, active edge, and inactive core), one-way ANOVA with Tukey's post-hoc test was used. The number of asterisks represents the p-value for the t-test and one-way ANOVA:  *p < 0.05, **p < 0.01, ***p < 0.001, ****p < 0.0001.

## Supplementary Information

Supplementary Information.

## Data Availability

Data supporting the results reported in this article are stored in the Yong laboratory, and the contact point person is VW Yong. All datasets will be made available to readers upon request. The MS brain spRNAseq datasets are available to download from the NCBI Sequence Read Archive (SRA) with BioProject accession number: PRJNA734097 upon publication of Dong et al., Nature Aging, in press, Feb 26 2022 (Dong Y, Jain RW, D’Mello C, Lozinski B, Visser F, Ghorbani S, Zandee S, Brown DI, Prat A, Xue M, Yong VW, Single cell and spatial RNA sequencing identify perturbators of microglia functions with ageing, Nature Aging, in press).

## References

[1] Reich DS, Lucchinetti CF, Calabresi PA (2018). Multiple sclerosis. N. Engl. J. Med..

[2] Tobore TO (2021). Oxidative/nitroxidative stress and multiple sclerosis. J. Mol. Neurosci..

[3] Yong HYF, Yong VW (2021). Mechanism-based criteria to improve therapeutic outcomes in progressive multiple sclerosis. Nat. Rev. Neurol..

[4] Tanaka M, Vecsei L (2020). Monitoring the redox status in multiple sclerosis. Biomedicines.

[5] Lassmann H, van Horssen J (1862). Oxidative stress and its impact on neurons and glia in multiple sclerosis lesions. Biochim. Biophys. Acta.

[6] Adamczyk B, Adamczyk-Sowa M (2016). New insights into the role of oxidative stress mechanisms in the pathophysiology and treatment of multiple sclerosis. Oxid. Med. Cell. Longev..

[7] Dong Y (2021). Oxidized phosphatidylcholines found in multiple sclerosis lesions mediate neurodegeneration and are neutralized by microglia. Nat. Neurosci..

[8] Haider L (2011). Oxidative damage in multiple sclerosis lesions. Brain.

[9] Nikic I (2011). A reversible form of axon damage in experimental autoimmune encephalomyelitis and multiple sclerosis. Nat Med.

[10] van Horssen J, Witte ME, Schreibelt G, de Vries HE (1812). Radical changes in multiple sclerosis pathogenesis. Biochim. Biophys. Acta.

[11] van Horssen J (2008). Severe oxidative damage in multiple sclerosis lesions coincides with enhanced antioxidant enzyme expression. Free Radic. Biol. Med..

[12] Chora AA (2007). Heme oxygenase-1 and carbon monoxide suppress autoimmune neuroinflammation. J. Clin. Invest..

[13] Holley JE, Newcombe J, Winyard PG, Gutowski NJ (2007). Peroxiredoxin V in multiple sclerosis lesions: Predominant expression by astrocytes. Mult. Scler..

[14] Hu CL (2019). Reduced expression of the ferroptosis inhibitor glutathione peroxidase-4 in multiple sclerosis and experimental autoimmune encephalomyelitis. J. Neurochem..

[15] Filippi M (2019). Association between pathological and MRI findings in multiple sclerosis. Lancet Neurol..

[16] Plemel JR (2020). Microglia response following acute demyelination is heterogeneous and limits infiltrating macrophage dispersion. Sci. Adv..

[17] Plemel JR (2018). Mechanisms of lysophosphatidylcholine-induced demyelination: A primary lipid disrupting myelinopathy. Glia.

[18] Keough MB, Jensen SK, Yong VW (2015). Experimental demyelination and remyelination of murine spinal cord by focal injection of lysolecithin. J. Vis. Exp..

[19] Jensen SK (2018). Multimodal enhancement of remyelination by exercise with a pivotal role for oligodendroglial PGC1alpha. Cell Rep..

[20] Glatigny S, Bettelli E (2018). Experimental autoimmune encephalomyelitis (EAE) as animal models of multiple sclerosis (MS). Cold Spring Harb. Perspect. Med..

[21] Hendrickx DAE (2017). Gene expression profiling of multiple sclerosis pathology identifies early patterns of demyelination surrounding chronic active lesions. Front. Immunol..

[22] Elkjaer ML (2019). Molecular signature of different lesion types in the brain white matter of patients with progressive multiple sclerosis. Acta Neuropathol. Commun..

[23] Fischer MT (2013). Disease-specific molecular events in cortical multiple sclerosis lesions. Brain.

[24] Yu Y (2021). Ferroptosis: A cell death connecting oxidative stress, inflammation and cardiovascular diseases. Cell Death Discov..

[25] Mendiola AS (2020). Transcriptional profiling and therapeutic targeting of oxidative stress in neuroinflammation. Nat. Immunol..

[26] Dong Y, Yong VW (2019). When encephalitogenic T cells collaborate with microglia in multiple sclerosis. Nat. Rev. Neurol..

[27] Brown D, Moezzi D, Dong Y, Koch M, Yong VW (2021). Combination of hydroxychloroquine and indapamide attenuates neurodegeneration in models relevant to multiple sclerosis. Neurotherapeutics.

[28] Faissner S (2018). Unexpected additive effects of minocycline and hydroxychloroquine in models of multiple sclerosis: Prospective combination treatment for progressive disease?. Mult. Scler..

