# Supplementary material for: Expression of antioxidant enzymes in lesions of multiple sclerosis and its models
Source: Sci Rep. 2022 Jul 26;12:12761. doi: 10.1038/s41598-022-16840-w (PMC9325863; doi:10.1038/s41598-022-16840-w)

Supplementary figures

Expression of antioxidant enzymes in lesions of multiple sclerosis and its models

Dorsa Moezzi[1]*, Yifei Dong[1]*, Rajiv W. Jain[1], Brian M. Lozinski[1], Samira Ghorbani[1], Charlotte D'Mello, and V. Wee Yong[1]**

Legend to Supplementary Figures:

Supplementary Fig 1: A, C, E) Representative confocal images of NAWM or POVPC-injured mice labeled with GFAP for astrocytes (white) and antioxidant enzyme of interest (red). Dotted line indicates the lesion ROI selected for image analysis. B, D, F) Bar graphs comparing the percent of ROI that is GFAP$^+$**,** and GFAP$^+$ antioxidant enzyme$^+$. Data are shown as mean ± S.D, n=6-12 mice. Significance indicated as ** $p< 0.01$, two-tailed, paired student's t-test.

Supplementary Fig 2: A, C, E) Representative confocal images of NAWM or LPC-injured mice labeled with GFAP for astrocytes (white) and antioxidant enzyme of interest (red). Dotted line indicates the lesion ROI selected for image analysis. B, D, F) Bar graphs comparing the percent of ROI that is GFAP$^+$**,** and GFAP$^+$ antioxidant enzyme$^+$. Data are shown as mean ± S.D, n=6 mice. Significance indicated as * $p< 0.05$, ** $p< 0.01$, two-tailed, paired student's t-test.

Supplementary Fig 3: A, C, E) Representative confocal images of NAWM or EAE-induced mice labeled with GFAP for astrocytes (white) and antioxidant enzyme of interest (red). Dotted line indicates the lesion ROI selected for image analysis. B, D, F) Bar graphs comparing the percent of ROI that is $GFAP^{+}$**,** and $GFAP^{+}$ antioxidant $enzyme^{+}$. Data are shown as mean ± S.D, n=4 mice. Significance indicated as * p< 0.05, two-tailed, paired student's t-test.

Supplementary Fig 4: Immunofluoresence of an MS tissue section of the white matter of the frontal lobe near (but not immediately adjacent) to the sections displayed in Figure 5. Section is stained for CD45 (green) and MBP (blue).

Supplementary Figure 1

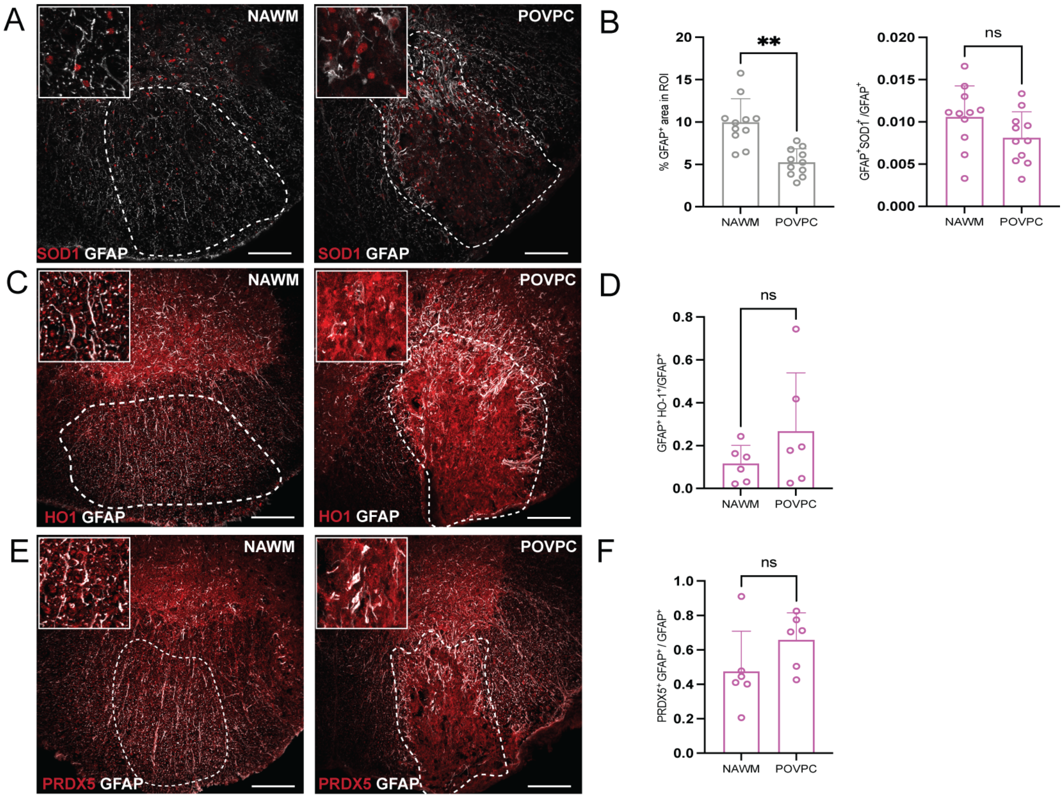

Supplementary Figure 2

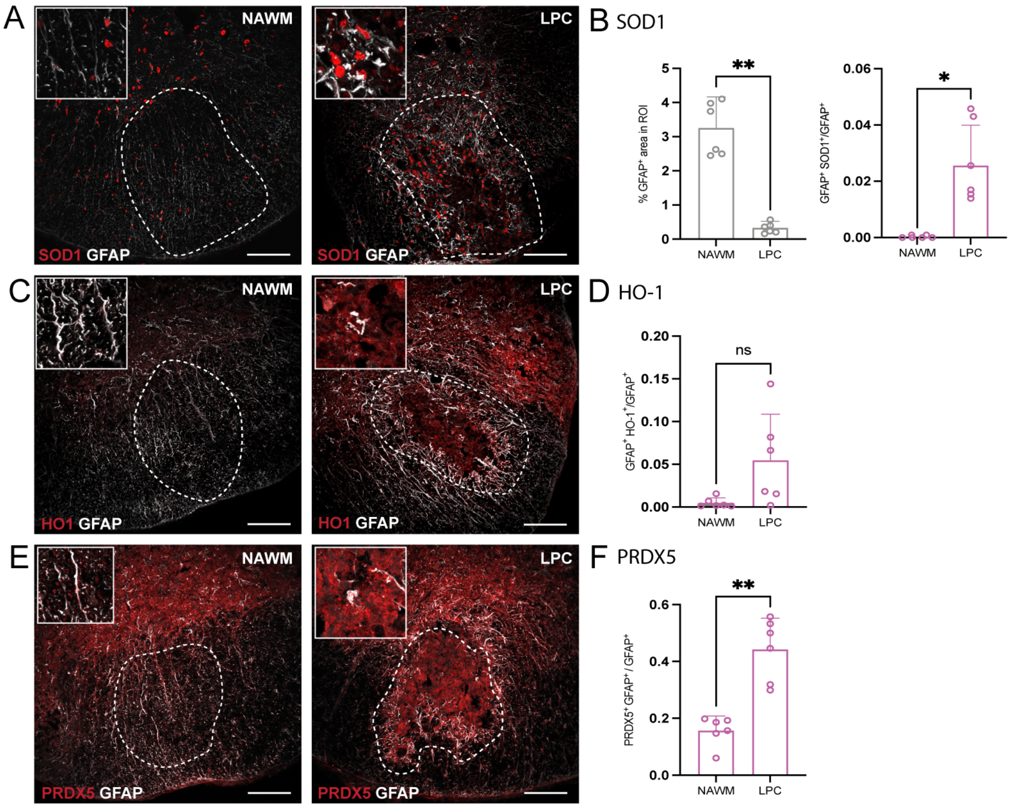

Supplementary Figure 3

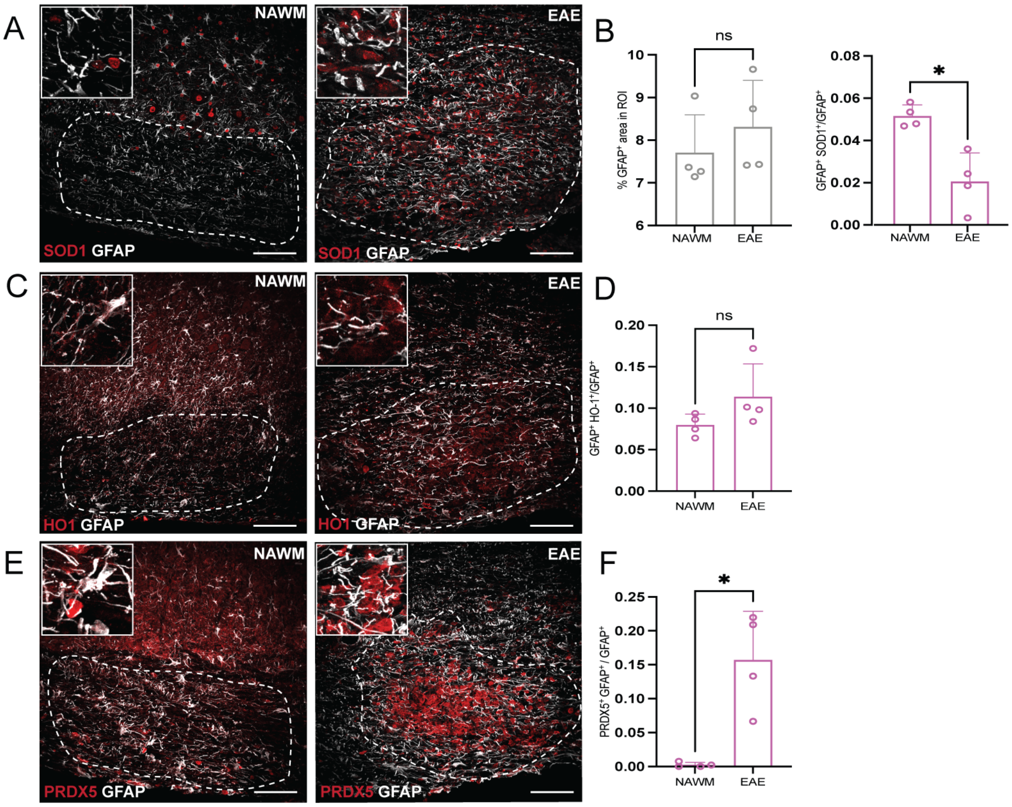

Supplementary Figure 4

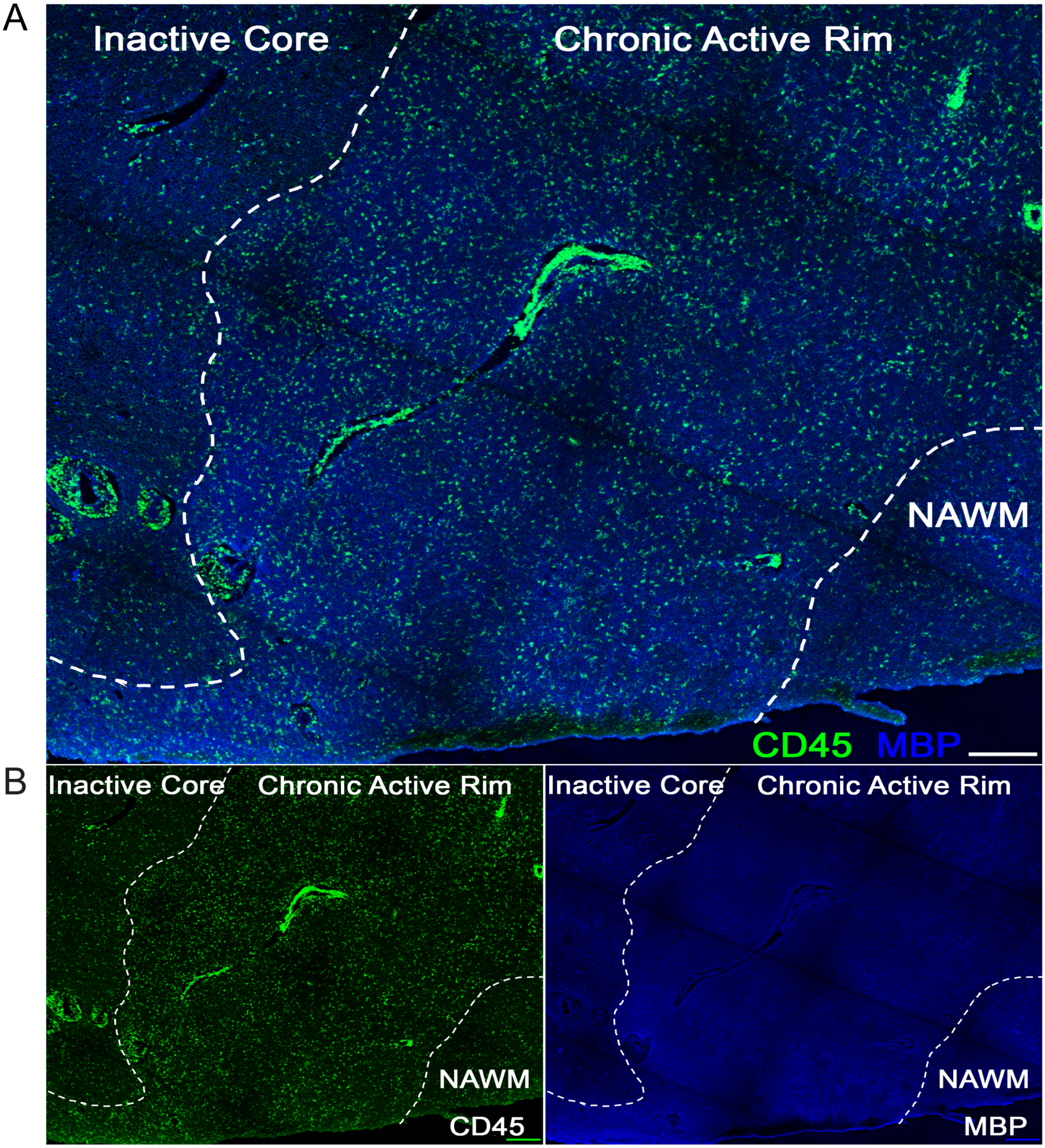

Supplement: Supplementary file 1 — Supplementary Information. [file 41598_2022_16840_MOESM1_ESM.pdf]
